# Nutrient Inadequacy in Korean Young Adults with Depression: A Case Control Study

**DOI:** 10.3390/nu15092195

**Published:** 2023-05-05

**Authors:** Su-In Yoon, Hye-Ri Moon, So Rok Lee, Jingnan Zhang, Soojin Lee, Jin Ah Cho

**Affiliations:** 1 Department of Food and Nutrition, Chungnam National University, Daejeon 34134, Republic of Korea; suinyoon@cnu.ac.kr (S.-I.Y.); mhyeri02108@o.cnu.ac.kr (H.-R.M.); sj807sr@cnu.ac.kr (S.R.L.); lkl1997@o.cnu.ac.kr (J.Z.); 2 Research Center for Microbiome-Brain Disorders, Chungnam University, Daejeon 34134, Republic of Korea; 3 Department of Microbiology and Molecular Biology, Chungnam National University, Daejeon 32134, Republic of Korea

**Keywords:** depressive symptoms, nutrient, nutrient adequacy, Korean, young

## Abstract

The role of diet is gaining attention among the modifiable factors associated with depression; thus, this case–control study examined the association between nutrition and depression in young Korean adults. Dietary surveys in individuals with depression (*n* = 39) and age- and gender-matched controls (*n* = 76) were conducted using food records and food frequency questionnaires. Men with depression consumed less mushrooms and meat, while women consumed significantly less grains (*p* < 0.05). Overall, the depression group consumed less energy and nutrients, and the difference was more pronounced in men. The male depression group had lower nutrient adequacy ratio (NAR) for energy, protein, vitamin A, thiamine, niacin, folate, and phosphorus, whereas the female depression group had lower NARs for energy, protein, niacin, and vitamin B_12_. The depression group had a significantly lower mean adequacy ratio in both genders. Furthermore, the proportion of inappropriate nutrient intake was higher in both genders of the depression group, exhibiting significant differences in energy, protein, niacin, folate, and zinc in men and energy, riboflavin, folate, and vitamin C in women. Hence, both men and women in the depression group had poor nutrient intake and high rates of nutrient inadequacy and improper consumption. This suggests that the quantity and quality of meals should be improved for individuals with depressive symptoms.

## 1. Introduction

Depression, along with bipolar disorder, schizophrenia, and obsessive compulsive disorder, is one of the most common mental disorders in many countries today and a serious public health concern. According to the World Health Organization (WHO), depression is a major cause of disability worldwide and a major contributor of the overall global disease burden affecting 280 million people of all ages worldwide (Institute of Health Metrics and Evaluation) [1]. 

The prevalence of depression among adults in Korea was estimated to be 5.6% in 2006, 6.7% in 2011, and 10.3% in 2013 [2]. In Korea, depression is on the rise, particularly among young adults. According to recent domestic health insurance data, hospital visits increased by 25% between 2019 and 2022, and the number of patients in their 20s increased by nearly 100%. Considering the global increase in depression by 50% over 27 years between 1990 and 2017, it disproves the rapid increase in depression among young people in Korean society [3]. This phenomenon can probably be explained by improved training for medical professionals and patients, changes in the way people view mental illness, COVID-19, etc. Although genetic and biological factors play a significant role in schizophrenia and bipolar disorder, which are considered severe mental illnesses, psychosocial factors, such as stress and environmental factors, play a significant role in depression. The recent increase in adolescent depression and the fact that suicide is the leading cause of mortality among those in their 10s and 30s in Korea are indicative of the environmental causes of this condition [4]. 

Although antidepressants are the most well-established form of treatment for depression, the response rate to them is about 60% [5]. Furthermore, the negative physiological effects and expenses associated with the use of prescribed antidepressants remain a problem [6,7]. As a result, the approach to treating depression focuses on preventing the onset of depression and developing effective self-leading wellness techniques rather than only symptoms of depression.

In recent years, nutrition has received much attention as a means of preventing illness and improving health. The link between nutrition and clinical depression and its symptoms is becoming increasingly apparent [8,9,10], and previous studies have reported that individuals’ dietary attitudes and nutritional status are related to the onset, intensity, and duration of depression [11]. Lack of appetite, skipping meals, and craving for sweet food can be examples. In the while, α-linolenic acid, folate, omega-3 unsaturated fatty acids, fruit and vegetable [12,13], and frequent fish consumption [14] all lowered the risk of depression [15]. Recent research has focused more on the relationship between the gut and the brain due to the discovery that the gut microbiome influences brain function and likely also mood and behavior, introducing a new way in which diet can affect mental health and disorders [16,17]. Nutritional neuroscience is a new field of study that demonstrates the relationship between nutritional factors and human cognition, behavior, and emotions via inflammation, oxidative stress, epigenetics, mitochondrial dysfunction, the gut microbiome, tryptophan-kynurenine metabolism, the HPA axis, neurogenesis and BDNF, epigenetics, and obesity [18,19]. 

Despite a dramatic increase in the proportion of depressed individuals and a very likely decrease in healthy behavior during the transition to adulthood [20,21], young adults remain a relatively neglected age group compared to children and older adults in diet-depression research. Moreover, a systematic review highlighted that just becoming an adult is a risk period for both low diet quality and poor mental health [22]. As a result of the provision of free meals to high schools in Korea, school nutritionists manage the quality and nutrition of meals, and as students enter maturity, the majority of their meals consist of individual decisions, revealing their vulnerability. Due to the lack of information on the relationship between dietary intake and depression in Korean young adults, the current study was carried out to ascertain the relationship between diet and depression by comparing the depressed group and age- and sex-matched controls with a focus on young adults in their 20s and 30s. 

## 2. Materials and Methods

### 2.1. Study Participants and Design

We conducted a case–control study of 19–39 young adults with and without depressive symptoms, matched 1:2 by age (±2 years) and sex. The study protocol was approved by the Ethical Committee of Chungnam National University in Daejeon, Korea [202012-SB-169-01]. Regarding ethical considerations, all subjects provided signed informed consent. The sample size was calculated using G-power V.3.1.9.4 (Heinrich-Heine-Universität Düsseldorf, Düsseldorf, Germany). A theoretically large effect size value of 0.8 was used to compare two independent means. With a power of 0.8 and a significance level of 0.05, the sample size calculation resulted in at least 26 individuals per group [23]. Finally, we enrolled 125 participants: 39 cases and 76 controls. 

Participants were recruited through hospital and campus-wide or social media marketing to participate in a depression study in Daejeon, Chungnam, Republic of Korea between April 2021 and December 2021. The Case was young adults (46% male) aged 19 to 39 years with depressive symptoms. The trained interviewer directly interviewed and evaluated depressive symptoms using the Korean-validated version of the Hamilton depression rating scale (K-HDRS). Depressed groups were categorized when they scored more than 17 points based on KHDRS. Those who use antidepressants were excluded. During the study period, 45 (men 19, women 26) subjects were identified with depressive symptoms; of which, 6 subjects who took antidepressants (*n* = 2) and gave up taking stool samples (*n* = 4) were excluded. A total of 39 subjects (men 18, women 21) had completed data on variables of interest. 

A total of 134 potential controls were contacted during hospital and campus-wide or social media marketing recruitment. The controls were those who scored 0 to 16 points without a history of depression in the past year on the KHDRS. A total of 76 controls (35 men, 41 women) with random matching for age (±2 years) and gender completed data collection for analysis. None of the variables were omitted from the responses of the 115 participants to the survey. In summary, the inclusion criteria were as follows: (1) young adults between the ages of 19 to 39; (2) a K-HDRS score ≥ 17 as assessed by the trainer; and (3) no antidepressant use. Moreover, the controls were those who scored 0–16 on the KHDRS and had no history of depression before. 

### 2.2. General Characteristics and Covariates

Approximately 40 min face-to-face interviews were conducted with each subject to collect data, including K-HDRS, lifestyle, and semi-quantitative dietary frequency questionnaires. Sociodemographic variables included sex, age (year), marital status (single or married), and education status (high school or college above). Health behaviors included smoking habits, exercise (none, 1–4/w or 5–7/w), supplementations intake, and probiotics intake. Except for exercise, all health behaviors covariates were dichotomized as yes or no. After completing all of the questionnaires, the study participants were instructed on how to assess the food and drinks that they consumed. They were asked to record estimated portion sizes of each ingested item and take a photo about it before and after eating. A visual guide was provided to improve the accuracy of portion size estimates. They were given a telephone number and social network service for information and support, which they could communicate to get help to settle any issues that arose while completing the food records. 

### 2.3. Dietary Assessment

Dietary intake was assessed by using food records over a three-day period. To calculate intakes of calories and macro- and micronutrients, the data from the food records were inputted into a can-pro 5.0 (The Korean Nutrition Society, Seoul, Korea, 2021). This software includes a list of some foods commonly available in Korea, to which other items can be added to the database. In this way, we were able to include the nutritional composition of packed foods taken from food labels. To investigate long-term diet effect, dietary intake also was calculated with an FFQ with 127 food items over the past 12 months, asking for the portion size of most items as described previously [24]. The validity and reliability of the FFQ in terms of nutrient and food consumption have been documented in detail in others [25]. The 127-line items on FFQ were collapsed into 20 food groups designed to replicate food groups created in a previous Agriculture department in Korea. Considering our subjects’ age category, we added a fast food group. This data was used to calculate food intake by food group and alcohol consumption. 

### 2.4. Estimate of Nutrient Adequacy/Deficiency

Korean Dietary Reference Intakes (KDRIs) include values of Estimated Average Requirements (EARs), Recommended Nutrient Intake (RNI), Adequate Intakes (AIs), Tolerable Upper Intake Levels (ULs), and Chronic Disease Risk Reduction Intake (CDRR), as well as Estimated Energy Requirements (EERs) for energy, and Acceptable Macronutrient Distribution Range (AMDR) for macronutrients. The percentage of energy provided by proteins, fats, and carbohydrates was also calculated and compared with AMDR.

To estimate the nutrient adequacy of the diet, the Nutrient Adequacy Ratio (NAR) was calculated for the energy intake and 14 nutrients (Table 5). The NAR for a given nutrient is the ratio of the subject’s intake to the current recommended nutrient intake for the subject’s sex and age category. NAR was truncated at 1 so that a nutrient with a lower NAR could contribute significantly to the average NAR for each group. The NAR of energy is based on the average daily energy requirements on the base of age and sex. As an overall measure of nutrient adequacy, the Mean Adequacy Ratio (MAR) was then calculated by averaging the NAR as described by Madden et al. (1976) [26]. This index quantifies the overall nutritional adequacy of a population based on an individual’s diet using the current recommended allowance for a group of nutrients of interest [27]. We have used the average of the 15 NARs mentioned in Table 5.

MAR (Mean Adequacy Ratio) = ∑NAR (each truncated at 1)/Number of nutrients.(1)

### 2.5. Anthropometric Measurements 

Height and weight were recorded by standing barefoot by the same person following standard procedures described by the WHO (in light clothing, without shoes, and having removed accessories, e.g., watches and wallets). Using a calibrated digital stadiometer, height and weight were measured to the nearest 0.1 cm and 0.05 kg, respectively. BMI was calculated as an index of obesity. Obesity was defined as above 25 according to WHO and NIH guidelines for Asians. Normal was defined below 25.

Body mass index (BMI) (kg/m^2^) = weight (kg)/height (m^2^). (2)

### 2.6. Assessment of Depressive Symptoms

HDRS, a 17-item inventory with scores ranging from 0 to 52, is the most widely used clinician-administered depression diagnostic scale. Increased depressive symptoms are indicated by higher scores. Each response to eight items was graded on a three-point Likert scale from 0 to 2 (0 = ‘Never’, 2 = ‘Very Often’), while the response to the last nine items was graded on a five-point Likert scale ranging from 0 to 4 (0 = ‘Never’, 4 = ‘Very Often’). The reliability of the Korean version of the HDRS was validated [28]. A K-HDRS score of 17 and above are regarded as indicative of “caseness” (depressed state) [28]. 

### 2.7. Statistical Analysis

Continuous variables are represented by means ± standard deviation (SD) or as medians (interquartile ranges (IQR)), while categorical variables are represented by frequency (percentage, %). The Kolomogorov–Smirnov test was performed to determine the normality of the examined variables’ distribution. The Student’s *t*-test (age, height, weight, BMI, KHDR score, and nutrient intakes) and, whenever appropriate, the non-parametric Mann–Whitney U test (alcohol intake, food group consumption, NAR, and MAR) were employed to compare the groups. To examine the relationship between categorical variables, the Chi-square test was performed. The significance level was established at *p* < 0.05 and statistical analysis was carried out using IBM SPSS version 26.0 software (SPSS Inc., Chicago, IL, USA).

## 3. Results

### 3.1. General Characteristics and Lifestyle Habits of the Participants

Table 1 listed the general characteristics of the participants, including 39 cases and 76 controls. In this study, case and control was one to two ratio sex and age matched, thus, their gender distribution and age were identical. Men accounted for 46.2% of the total participants. In men and women, both depressive and non-depressive groups have similar physical and sociodemographic features. Their height, weight, and BMI did not show statistical differences. Marital status and educational background were very similar, both were highly educated and most of them were single. 

In the health behavior-related factors, the female depression group consumed slightly more alcohol compared to normal (*p* = 0.066). On the basis of the FFQ, the daily alcohol consumption was estimated by multiplying the amount by the value converted by the frequency of drinking per day. For the FFQs, the ml of alcohol consumed by each beverage type (beer, wine, rice wine, etc.) was multiplied by a factor expressing the proportion of alcohol contained in each beverage, and this value was confirmed through each company’s nutritious labeling. The ml of daily average alcohol consumption was converted into grams. In addition, the female depression group had a higher portion of smokers compared to the normal group (14.3% vs. 0.0%, *p* = 0.013). Meanwhile, in the male depression group, although proportion of subjects did not exercise at all was higher (27.8%) compared to normal group (17.1%), the frequency of more than five times per week was significantly lower in cases (0.0%) than in controls (37.1%, *p* = 0.012) which is consistent with previous research indicating that individuals with depression showed decreased physical activity [29,30,31]. 

### 3.2. Nutrient and Food Intake According to Depressive Symptoms and Gender

Table 2 compares absolute intake (95% CI) and effect size of macronutrients and micronutrients between participants with and without depressive symptoms stratified by gender. In men, most nutrients, including total energy, carbohydrate, protein, fat, vitamins A, C, E, thiamin, niacin, folate, phosphorous, zinc, and selenium (*p* < 0.05) showed a statistically significant difference between the two groups all of which were lower in depression group. In women, there was significant differences in energy, carbohydrate, protein, niacin, vitamin B_12_ and C, phosphorous, and selenium, and in all of these, intake amount of the depression group was lower than that of the normal group. Total energy intake was 1821 ± 552 kcal/day and 1550 ± 496 kcal/day for men and women, respectively, in the depressed group, and 2401 ± 611 kcal/day and 1807 ± 404 kcal/day for men and women in the non-depressive group. Men and women in the depression group consume 24.2% and 14.2% less energy than those in the normal group, respectively (*p* < 0.001). This shows that the overall food intake was lower in the depression group, and the decrease in energy intake in the male depression group was more pronounced compared to women (Table 2).

However, there was no difference in nutrient density (amount of nutrient intake /1000 kcal) of each nutrient adjusted for calories between depression and normal group in both men and women (data are not shown). Although the depression group seems to consume less of almost all the nutrients than the normal group, our data showed that people with depressive symptoms have a pattern of consuming less calories in general rather than a deficiency of a few specific nutrients.

However, in both groups, the proportion of calories obtained from carbohydrates, proteins, and fats was similar between the two groups. The 2020 Korean Dietary Guideline recommends an ADMR for carbohydrates (55–65% of energy), fats (15–30% of energy), and proteins (7–20% of energy) [32]. Interestingly, both groups have substantially lower carbohydrate ranges and higher fat ranges (Table 2), indicating that the dietary habits of the young generation in Korea have changed dramatically and become more westernized over the past decade.

Table 3 compares the consumption of food groups based on the presence of depressive symptoms by gender. Using data collected from three-day food records, the average number of servings consumed from each food group was calculated. Foods were grouped into sixteen food groups based on the food grouping as indicated by Korean Rural Development Administration (KRDA). The data distribution was not normal, so the median value was employed to represent the data and the Mann–Whitney test was used to evaluate statistical significance. The depression group consumed fewer fats and oils groups (*p* < 0.05). In addition, men in the depression group consumed significantly less meats and mushrooms (*p* < 0.05) and slightly less grains (*p* = 0.067). Women in the depression group, on the other hand, consumed less grains than normal (*p* < 0.05).

Using FFQ, we also compared the long-term dietary intake of the depressive symptom group to that of the normal group (Table 4). The 127 food lists presented in FFQ were categorized into 20 food groups based on the KRDA’s food classification. Considering the age of participants, the fast food group containing hamburgers and pizza was added, and the grain group was subdivided into whole grains, rice, and noodles. The male depression group consumed more sweets than the control group (*p* = 0.011), while the female depression group consumed less rice (*p* = 0.008), noodles (*p* = 0.025), and soda (*p* = 0.008) than the control group.

### 3.3. Nutritional Adequacy Assessment According to Depressive Symptoms and Gender

Table 5 shows the NAR and MAR of participants classified by depressive symptoms and gender. The NAR for protein, thiamin, vitamin B_12_, phosphorus, and iron was close to one in almost all groups regardless of depressive symptoms or gender. In contrast, the NAR for vitamins A, C, and calcium was 0.31–0.61, 0.34–0.61, and 0.48–0.65, respectively, indicating inadequate intake.

The NAR of energy, protein, and niacin was significantly lower in both men and women in the depression group than in the control group (*p* < 0.05). Interestingly, the values of folic acid in the male depression group and vitamin B_12_ in the female depression group were significantly lower than in the control group (*p* < 0.05).

Overall, the median MARs for men and women with depressive symptoms were 0.75 (0.65–0.88) and 0.80 (0.64–0.92), respectively, and those in controls were 0.86 (0.78–0.93) and 0.88 (0.80–0.94), respectively. There was a significant difference between the depressive group and the normal group (*p* = 0.009 and 0.056). The ideal cutoff for nutrient adequacy is one, indicating that all nutrients are adequately consumed. This level was not attained by any of the respondents in this study. Furthermore, only 51.3% and 84.2% of the depressive and normal groups had MAR values greater than 0.75. The minimum appropriate level was determined to be 0.75 of MAR.

Table 6 shows the proportion of the subjects taking less than 75% of the EAR for each nutrient, based on the presence of depressed symptoms, stratified by gender. For energy, the estimated energy requirement has superseded the EAR. Regarding micronutrients, in men, inadequate intakes (i.e., over 20% of participants below the age and sex-specific EAR thresholds (75% of the EAR)) were observed for vitamin A, folic acid, vitamin C, and calcium in both depressed and normal groups. Furthermore, the proportion of subjects with less than 75% of the EAR for the aforementioned nutrients was greater than 50% in the depressed group. In women, a similar pattern was observed. Regarding energy, energy intake inadequacy proportion was observed much higher in the depressive group in both genders (55.6%, 22.9%, 42.9%, and 17.1% for with and without depressive symptoms of men and women, respectively).

In the group with depressive symptoms, the proportion of individuals having inadequate intakes of energy, protein, folic acid, and zinc in men and of energy, riboflavin, folic acid, and vitamin C in women was significantly higher. The average intake for the majority of nutrients was found to be almost consistently greater for males compared to females; however, the percentages of males with inadequate dietary intakes were found to be higher (Table 2 and Table 6).

## 4. Discussion

This study examines whether overall nutritional adequacy is the factor to distinguish the absence or presence of depressive symptoms in 20–30s young adults. The findings of the current study indicate that the depressive symptom group had poor energy and nutrient intake, insufficient nutritional adequacy, low MAR values, and a high proportion of each nutrient deficiency. Our study is preliminary as it was limited by its small size and cross-sectional design. The link between diet and depression is still unexplained; therefore, further studies are warranted to investigate the potential relationship between the dietary-affected microbiome or inflammation markers in serum and depression in this population.

Due to the prevalence of depression and the enormous economic burden it causes, it is crucial to evaluate risk factors, such as food. Recent epidemiological research has connected diet as a modifiable factor to oxidative stress [33,34,35], inflammation [36,37,38,39], as well as brain plasticity and function [40]. All of these factors have a substantial impact on an individual’s mental health and the onset of depression [19]. The relationship between unhealthy diet and depression can be justified in this way. High consumption of processed and red meats was associated with elevated levels of low-grade inflammation (C-reactive protein) and pro-inflammatory adipokine (leptin) as well as subsequent brain atrophy which is positively correlated with depression [41,42]. In addition, excessive intakes of sugar, fast food, snacks, and soft drinks were associated with depression due to direct changes in gut microbiota functions and microbial metabolism which have the potential to affect the neural network [43]. In contrast, a nutritious diet can have the opposite effect.

Due to the complex interaction between nutrients or foods in a diet and mental health, a single examination may not provide a complete picture of the relationship. Thus, analysis of food patterns has recently garnered a great deal of interest among researchers [44,45,46]. Certain diets, such as the Mediterranean diet, have been linked to better mental health in adults [47,48,49]. Our investigation was unable to distinguish between the two groups’ food patterns, including dietary inflammatory index [50]. These inconsistencies may result from small sample sizes in depressive groups, different populations, and insufficient intensity of depression to distinguish between the two conditions. However, our study clearly revealed that the absolute amount of food consumption in the depression group was less than the control group.

Depression exhibits the opposing symptoms of increased and decreased appetite on occasion [51,52,53]. It was confirmed that the activated brain regions were different due to differences in these symptoms. Depression-related appetite loss was associated with reduced nucleus accumbens (NAcc) based functional connectivity to the ventromedial prefrontal cortex (vmPFC) and the hippocampus [54] and hypoactivation of the insular region [55] both of which are associated with reward and interoceptive neurocircuitry. According to several studies, appetite loss is a more significant symptom of depression in adolescents than appetite rise [56,57]. In our study, the depression group among young adults demonstrated a reduction in total calorie and nutrient consumption, possibly due to decreased appetite. Therefore, the low BMI of the depression group, although it is not statistically significant, is likely due to appetite reduction.

Numerous individual nutrients, including vitamin B complex, vitamin D, choline, iron, zinc, magnesium, S-adenosyl methionine, taurine, and probiotics, have been investigated for their effects on depression. There is substantial evidence of the efficacy of B vitamins to support the use of combination high-dose B vitamins in the treatment of stress and anxiety in the general population [58].

Folic acid and vitamin B_12_ are required for the synthesis of S-adenosylmethionine which functions as an essential methyl donor in a number of crucial methylation activities in the central nervous system. When S-adenosylmethionine synthesis is blocked, DNA methylation and the generation of monoamine-based neurotransmitters, such as serotonine and catecholamines, may be reduced [59]. Deficiency in either folic acid or vitamin B_12_ results in neurological or psychiatric disease [60,61,62,63]. Practically, folic acid and vitamin B_12_ deficiencies are common among patients suffering from neuropsychiatric disease [64]. However, the relationship between the dietary intake of folate or vitamin B_12_ and the risk of depression has not been well established [65,66,67].

The male depression group had lower folic acid intake and NAR than the normal group with more than 50% taking less than 75% of the EAR. This indicates that men’s folic acid consumption is inadequate. In the female depression group, there was no difference between folic acid consumption and NAR; however, less than 75% of EAR intake was higher in the female depression group (42.9% vs. 19.1%, *p* = 0.051). In this study, it is not possible to identify whether low folic acid consumption is the cause or effect of depression, but it is acceptable to believe that a low folic acid intake in conjunction with a poor nutrient intake can intensify or prolong depression at least in men [68].

Vitamin B_12_, along with folic acid, plays a crucial part in the single carbon transfer reaction and is widely associated with depression. However, vitamin B_12_ insufficiency is not prevalent among Koreans. Similarly, this study showed that there was no difference between the male depression group and the normal group in terms of vitamin B_12_ intake and NAR. In addition, it was uncommon for either group, regardless of the level of depression, to consume vitamin B_12_ less than 70% of EAR. This is likely attributable to the meat-eating pattern of men. However, for women in the depression group, NAR levels and vitamin B_12_ intake were low, although a few individuals in both groups consumed less than 75% of EAR. Although vitamin B_12_ insufficiency is uncommon, insufficient nutrition caused by depression might exacerbate the condition in women who consume less meat.

Interestingly, folate deficiency in men and vitamin B_12_ deficiency in women were also observed in prior research [69]. This demonstrates that a gender-based intervention is required if nutrition is associated with the onset of depression.

In our study, we used three days’ estimated food records to assess nutritional status. The limitations of the weighed records are said to be under reporting, under-representation of ‘usual diet’ over an insufficient number of days and distortion of food habits due to the recording process [70]. To circumvent these restrictions, food consumption was calculated based on portion sizes learned from an initial interview. The data collection was rigorously supervised, and methodological difficulties were frequently reviewed throughout the duration of the study. To encourage and support, we utilized an online social networking platform to swap photographs before and after meals.

Accurate measurement of alcohol intake is crucial for valid estimation of alcohol related effects in general epidemiology study and is especially necessary in depressed patients. Alcohol problems in depression is more common than in general population and is associated with worse outcomes with respect to depression course, suicide/death risk, social functioning, and health care utilization [71]. In our study, the mean alcohol consumption for depressive group was 12.6 ± 21.9 g/day (around 12 g alcohol/standard one glass) which is light drinkers’ level even though higher than control group. A longitudinal population-based study in Sweden, non-drinker had a higher depression risk than light drinkers, but only hazardous drinking was associated with a higher risk of depression (risk ratio: 1.8, 95% confidence internal: 1.4–2.4) [72]. Based on this study, our depressive group and non-depressive group both do not have issues with alcohol consumption.

Previous study shows that food records and FFQ show differences in the recorded prevalence of drinking and amount of consumed alcohol despite moderate agreement between the two methods. In our study, the mean daily alcohol intake of consumers was 11.05 ± 21.45 g/day and 5.56 ± 15.05 g/day, as assessed by the FFQ and the DR, respectively. Alcohol intake difference was shown between the depressive symptom group and control only by FFQ not food records. We collected the food records only for three days which is probably insufficient to cover daily alcohol consumption and seems to underestimate alcohol consumption. More detailed alcohol-specific questionnaires [73,74] would be required to obtain more precise alcohol intakes.

The food group intake results also differed slightly depending on the dietary survey methodology. An evaluation of food group intake based on the results of the dietary records showed that men in the depression group had less meat and mushrooms, while women in the depression group had less grain intake. Low fats and oil consumption have been observed in both male and female depression groups. Conversely, as a result of comparing long-term food intake using FFQ data, sugar intake was significantly higher in the male depression group, while rice, noodles, and beverage intake were significantly lower in the female depression group. The decrease in grain consumption was found in both investigative methods for female depression groups. This indicates that women’s grain consumption has decreased significantly in both long and short-term diets. On the other hand, in the male depression group, the food group that differed in short-term and long-term intake was different. This does not rule out the possibility that the actual short- and long-term results stem from differences in diet, that the short-term survey did not accurately reflect the daily intake or that the long-term intake survey included memory errors. It can also be thought that the correlation between long-term eating and depression has decreased because the onset of depression has not been investigated. Since FFQ investigates the consumption of a predetermined food list, the possibility of overestimating food intake should be considered, as investigation time is significant and depressive patients tend to take longer. In the case of FFQ, some point out that it will be beneficial to develop a simplified and specialized survey tool for depression subjects in the future.

Numerous studies have demonstrated that physical activity has preventative and therapeutic effect on depression [75,76,77,78]. In addition, the exercise would be an alternative to drugs or synergic effects, especially for persons who are not drug sensitive or who are healthy [79]. There is no need to highlight the importance of exercise. Our research also revealed that the depressed group engages in less physical exercise. The ratio of those who did not exercise to those who did exercise was comparable, but the number of subjects who exercised frequently was much lower in the depressive symptom group compared to the control. As interest in the use of exercise as a treatment for depression grows, the optimal program variables, including frequency, intensity, duration, and kind of exercise, are necessary [80,81].

The Korean National Health and Nutrition Examination Survey (KNHANES) is an annual nation-wide survey conducted by the Korea Centre for Disease Control and Prevention (KCDC) that provides essential data for national policies to enhance public health. Numerous research findings using these data have been published, and research on depression is currently being undertaken. Using the KNHANES V–VII (2010–2018) data, it has been reported that depression is associated with low intake of fruits and vegetables [82], low consumption of curry rice [83], seaweed and mushroom [84], sugar-sweetened beverages consumption (≥ 1/day) [85], short sleep (≤ 6 h) [86], increased work-related physical activity, decreased recreational physical activity [84,87], poor dietary habits [88,89], gender difference, night eating, and nutritional adequacy [90] in the general population.

On the other hand, in the case of depression with other diseases, lack of niacin in patients with metabolic syndrome [2], lack of omega-3 fatty acids in menopause women [91], and lack of vitamin D in patients with chronic kidney disease [92] were also reported. However, the majority of research has been conducted on adults aged 19 or older, and they compare subjects already diagnosed with depression and non-depressed people. Although our study is not a large-scale study, the fact that we focused on diet-related factors according to depression scores of subjects who have not yet been diagnosed with depression suggests that there is a difference in diet and nutritional quality even if the degree of depression is not severe and that a decrease in diet in young adults may be a sign of depression.

There are limitations to this study. The exclusion criteria (antidepressant drug taker, abandoners) may have resulted in underestimation of effect sizes and study results. The majority reason for giving up the study was failure to collect stool which was collected to investigate microbiome analysis. Those people may have a more severe dietary pattern or nutritional issue. Reverse causality and residual confounding cannot be fully excluded owing to the cross-sectional design, as temporal sequences between exposure and outcome cannot be established. The timing of the investigation coincides with international disasters, such as COVID-19; therefore, it cannot be ruled out that the depression experienced by young people may not coincide with general depression. To elucidate the relationship between diet and depression and determine what diet affects depression additional research is required.

In spite of this, our study detected inadequate nutritional status in depression groups of young adults in their 20s and 30s and validated gender-based disparities in the food and nutrients that were insufficient. For young Koreans who are exposed to rapid dietary vulnerabilities in adulthood, where poor eating habits can contribute to a worsening of the degree of depression and the persistence of its symptoms, efforts should be made to choose a better meal by employing potential tools, such as social media [73,74], nutritional education, and nutrition labeling [75].

## Figures and Tables

**Table 1:** The general characteristics of all the participants.

		Men			Women	
	Depressive	Normal	*p*-Value ^1^	Depressive	Normal	*p*-Value ^1^
	(*n* = 18)	(*n* = 35)		(*n* = 21)	(*n* = 41)	
Age (year)	23.0 ± 4.1 ^2^	24.2 ± 3.5	0.269	24.7 ± 4.8	25.8 ± 5.1	0.398
Height (cm)	172.3 ± 5.9	175.2 ± 5.4	0.074	162.0 ± 6.6	161.8 ± 5.0	0.884
Weight (kg)	71.9 ± 13.7	78.2 ± 12.5	0.102	56.9 ± 10.3	59.2 ± 10.6	0.400
BMI (kg/m^2^)	24.1 ± 3.9	25.4 ± 3.7	0.242	21.7 ± 3.7	22.6 ± 4.0	0.362
<25	11 (61.1) ^3^	13 (37.1)	0.097	15 (71.4)	28 (68.3)	0.800
≥25	7 (38.9)	22 (62.9)		6 (28.6)	13 (31.7)	
Sociodemographic factors						
Marital status (single)	17 (94.4)	32 (91.4)	0.694	18 (85.7)	33 (80.5)	0.610
Education level						
(More than College)	18 (100.0)	30 (85.7)	0.092	21 (100.0)	40 (97.6)	0.471
Health behavior factors						
Smoking (yes)	7 (38.9)	12 (34.3)	0.741	3 (14.3)	0 (0.0)	0.013
Probiotics (yes)	4 (22.2)	10 (28.6)	0.620	6 (28.6)	17 (41.5)	0.320
Supplements (yes)	2 (11.1)	5 (14.3)	0.746	5 (23.8)	5 (12.2)	0.239
Alcohol intake (g/day)	8.6 (1.5–26.6) ^4^	2.2 (0.0–19.7)	0.283 ^5^	0.7 (0.0–4.1)	0.0 (0.0–3.6)	0.066 ^5^
Exercise						
None	5 (27.8)	6 (17.1)	0.012	8 (38.1)	18 (43.9)	0.245
1–4/w	13 (72.2)	16 (45.7)		13 (61.9)	19 (46.3)	
5–7/w	0 (0.0)	13 (37.1)		0 (0.0)	4 (9.8)	
K-HDRS ^6^ score	20.4 ± 2.9	6.9 ± 5.2	<0.001	23.8 ± 5.1	6.9 ± 4.8	<0.001

^1^ Chi-Square test is used for categorical variables and *t*-test is used for continuous values. ^2^ Mean ± SD. ^3^ N (%). ^4^ Values are Median. The number in the bracket indicates first and third quartile. ^5^ Data were not normally distributed; non-parameter analysis was used to test statistical significance. ^6^ K-HDRS; Korean-Hamilton Depression Rating Scale, higher scores indicate stronger depressive symptoms.

**Table 2:** Nutrient intake of participants in relation to depressive symptoms by gender.

		Men				Women		
	Depressive	Normal	Effect	*p*-Value ^2^	Depressive	Normal	Effect	*p*-Value ^2^
Nutrient Intake	(*n* = 18)	(*n* = 35)	Size ^1^		(*n* = 21)	(*n* = 41)	Size ^1^	
Energy (kcal)	1821 ± 552 (1546, 2096) ^3^	2401 ± 611 (2191, 2611)	−0.73	0.001	1550 ± 496 (1323, 1775)	1807 ± 404 (1680, 1935)	−0.59	0.032
Carbohydrate (g)	208.8 ± 70.1 (173.9, 243.6)	256.4 ± 62.9 (234.8, 278.0)	−0.91	0.015	197.4 ± 68.0 (166.5, 228.3)	236.0 ± 51.7 (219.6, 252.3)	−0.67	0.015
Protein (g)	77.5 ± 32.0 (61.5, 93.4)	101.8 ± 32.7 (90.6, 113.0)	−0.75	0.013	59.3 ± 19.8 (50.3, 68.4)	70.6 ± 21.5 (63.8, 77.4)	−0.48	0.049
Fat (g)	66.3 ± 25.2 (53.8, 78.9)	95.8 ± 32.7 (83.7, 107.9)	−0.55	0.003	51.0 ± 20.3 (41.8, 60.2)	60.6 ± 20.0 (54.3, 66.9)	−0.54	0.079
Fiber (g)	13.8 ± 6.4 (10.6, 16.9)	17.7 ± 7.3 (15.1, 20.2)	−0.60	0.062	14.2 ± 7.7 (10.7, 17.7)	17.9 ± 7.9 (15.4, 20.4)	−0.47	0.082
Vitamin A (μg RAE)	283.5 ± 179.2 (194.4, 372.6)	438.8 ± 290.6 (339,538.7)	−0.60	0.044	435.3 ± 400.4 (253.0, 617.6)	470.0 ± 269.0 (385.1, 554.9)	−0.11	0.687
Thiamin (mg)	1.7 ± 0.7 (1.4, 2.1)	2.6 ± 1.7 (2.0, 3.2)	−0.56	0.045	1.5 ± 0.7 (1.1, 1.8)	1.6 ± 0.6 (1.4, 1.8)	-	0.397
Riboflavin (mg)	1.3 ± 0.5 (1.1, 1.6)	1.6 ± 0.6 (1.4, 1.8)	−0.73	0.060	1.3 ± 0.5 (1.1, 1.5)	1.5 ± 0.5 (1.3, 1.6)	−0.54	0.169
Niacin (mg)	14.0 ± 6.5 (10.8, 17.2)	19.6 ± 8.1 (16.8, 22.4)	−0.22	0.015	10.6 ± 4.2 (8.7, 12.5)	14.0 ± 5.2 (12.3, 15.6)	0.09	0.012
Vitamin B_6_ (mg)	1.8 ± 1.0 (1.3, 2.3)	2.0 ± 0.8 (1.8, 2.3)	−0.65	0.443	1.5 ± 1.3 (0.9, 2.1)	1.8 ± 1.4 (1.3, 2.2)	−0.08	0.513
Folic acid (μg)	276.3 ± 118.9 (217.2, 335.4)	367.1 ± 150.1 (315.6, 418.7)	−0.39	0.030	331.5 ± 187.2 (246.3, 416.7)	358.2 ± 139.3 (314.2, 402.2)	−0.16	0.528
Vitamin B_12_ (μg)	5.6 ± 4.2 (3.5, 7.7)	7.9 ± 6.5 (5.7, 10.2)	−0.62	0.183	4.8 ± 3.8 (3.1, 6.5)	7.6 ± 4.1 (6.3, 8.9)	−0.40	0.012
Vitamin C (mg)	33.7 ± 18.1 (24.7, 42.7)	57.8 ± 45.6 (42.1, 73.5)	−0.80	0.009	68.5 ± 108.0 (19.4, 117.7)	74.8 ± 63.1 (54.9, 94.7)	−0.70	0.774
Vitamin E (mg)	11.6 ± 6.6 (8.4, 14.9)	18.1 ± 8.8 (15.1, 21.1)	−0.41	0.008	12.1 ± 5.4 (9.6, 14.6)	15.3 ± 6.1 (13.3, 17.2)	−0.22	0.048
Vitamin K (μg)	62.3 ± 44.2 (40.4, 84.3)	89.7 ± 76.1 (63.6, 115.9)	−0.30	0.166	100.3 ± 105.7 (52.1, 148.4)	93.7 ± 52.6 (77.1, 110.3)	−0.17	0.743
Calcium (mg)	387.6 ± 172.6 (301.7, 473.4)	441.9 ± 187.4 (377.5, 506.2)	−0.86	0.310	430.0 ± 186.0 (345.5,514.4)	487.7 ± 195.1 (426.1, 549.2)	−0.70	0.267
Phosphorus (mg)	945.8 ± 354.2 (769.7, 1122.0)	1273.8 ± 396.3 (1137.7, 1410.0)	−0.39	0.005	845.2 ± 295.9 (710.5, 979.8)	1022.9 ± 303.4 (927.1, 1118.6)	−0.30	0.032
Iron (mg)	14.8 ± 5.6 (12.0, 17.6)	17.5 ± 7.8 (14.9, 20.2)	−0.68	0.187	13.3 ± 6.9 (6.3, 9.6)	14.4 ± 4.7 (8.0, 10.0)	−0.59	0.458
Zinc (mg)	10.2 ± 4.9 (7.7, 12.6)	13.3 ± 4.5 (11.8, 14.9)	−0.71	0.024	7.9 ± 3.7 (6.3, 9.6)	9.0 ± 3.1 (8.0, 10.0)	−0.55	0.227
Selenium (μg)	68.1 ± 27.8 (54.3, 81.9)	93.3 ± 39.1 (79.9, 106.8)	-	0.018	54.7 ± 25.4 (43.1, 66.2)	71.9 ± 28.0 (63.1, 80.7)	-	0.021
C: P: F ratio ^4^								
Carbohydrate (%)	45.9 ± 7.3	43.3 ± 7.0		0.211	51.2 ± 8.1	52.6 ± 6.2		0.444
Protein (%)	16.8 ± 4.3	16.9 ± 3.2		0.118	15.6 ± 2.9	15.6 ± 3.9		0.937
Fat (%)	32.6 ± 6.0	35.6 ± 6.6		0.932	29.6 ± 6.2	29.9 ± 5.5		0.835

^1^ mean difference/pooled SD. ^2^ *p* values from *t*-test. ^3^ Values are Mean ± SD, the number in the bracket indicates 95% CI. ^4^ (Energy from carbohydrate, protein or fat/total energy intake) × 100.

**Table 3:** Consumption of food groups in participants based on depressive symptom with genders from food records.

		Men			Women	
	Depressive	Normal	*p*-Value ^2^	Depressive	Normal	*p*-Value ^2^
Food Group ^1^	(*n* = 18)	(*n* = 35)		(*n* = 21)	(*n* = 41)	
Grains	2.11 (1.51–2.60) ^3^	2.62 (1.92–3.29)	0.067	1.97 (1.34–2.34)	2.47 (1.82–3.00)	0.029
Potatoes	0.04 (0.00–0.08)	0.07 (0.01–0.13)	0.174	0.03 (0.00–0.09)	0.04 (0.00–0.13)	0.498
Sweets	0.65 (0.31–0.92)	0.47 (0.17–1.20)	0.862	0.61 (0.20–1.39)	0.97 (0.40–1.67)	0.158
Legumes	0.06 (0.00–0.38)	0.11 (0.00–0.36)	0.659	0.17 (0.00–0.45)	0.15 (0.00–0.49)	0.981
Nuts	0.01 (0.00–0.10)	0.03 (0.00–0.22)	0.468	0.02 (0.00–0.12)	0.07 (0.00–0.15)	0.180
Vegetables	2.45 (1.75–4.48)	3.31 (2.06–5.18)	0.186	3.57 (1.61–5.15)	3.36 (2.61–4.46)	0.682
Mushrooms	0.00 (0.00–0.00)	0.00 (0.00–0.16)	0.033	0.03 (0.00–0.28)	0.00 (0.00–0.31)	0.441
Fruits	0.08 (0.00–0.40)	0.27 (0.01–1.01)	0.194	0.27 (0.06–1.14)	0.68 (0.11–1.18)	0.399
Meats	2.99 (2.07–4.57)	4.31 (2.86–6.55)	0.039	1.79 (1.24–2.49)	1.87 (0.76–2.77)	0.885
Eggs	0.25 (0.04–0.69)	0.51 (0.09–0.95)	0.278	0.39 (0.15–0.88)	0.56 (0.18–1.16)	0.412
Fishes and shellfishes	0.15 (0.00–0.70)	0.49 (0.05–1.36)	0.153	0.56 (0.15–1.12)	0.70 (0.41–1.40)	0.284
Seaweeds	0.19 (0.00–0.60)	0.06 (0.00–0.52)	0.618	0.00 (0.00–0.33)	0.10 (0.00–0.67)	0.137
Dairy products	0.20 (0.00–0.96)	0.22 (0.00–0.81)	0.735	0.70 (0.16–1.33)	0.78 (0.28–1.28)	0.831
Fats and oils	0.91 (0.37–2.08)	1.98 (0.91–3.17)	0.026	0.99 (0.49–2.03)	1.88 (1.20–2.23)	0.042
Drinks	1.08 (0.82–3.12)	1.98 (1.04–3.60)	0.140	1.19 (0.49–3.19)	1.31 (0.77–2.08)	0.855
Condiment	1.51 (0.92–2.25)	1.81 (1.06–3.19)	0.205	1.77 (1.08–2.67)	2.14 (1.29–2.84)	0.354

^1^ Foods were grouped into sixteen food groups based on the food grouping as indicated by Korean Rural Development Administration. ^2^ *p* values from Mann–Whitney U test. ^3^ Values are Median. The number in the bracket indicates first and third quartile. Median value of the serving size of the consumed food group has been adapted to the food intake recommendations and practices followed in Korea National Health and Nutrition Examination Survey (KNHNES). One serving size of grains and potatoes group equals approximately 300 kcal. For protein-based foods, such as meats, eggs, nuts, fish and shellfishes, one serving equals 100 kcal. Fruits have a 100 kcal/ 50 g serving size. Fruit juice was included in the category of fruits. Vegetables have 15 kcal, 70 g per serving size. One serving of drinks is 200 mL. Dairy products have a 125 kcal serving size. A total of 1 teaspoon of fats and oils equals approximately 45 kcal.

**Table 4:** Consumption of food groups of participants based on food frequency questionnaire.

		Men			Women	
	Depressive	Normal	*p*-Value ^2^	Depressive	Normal	*p*-Value ^2^
Food Group ^1^	(*n* = 18)	(*n* = 35)		(*n* = 21)	(*n* = 41)	
Whole grains	0.10 (0.03–0.84) ^3^	0.14 (0.00–1.00)	0.977	0.07 (0.00–0.50)	0.32 (0.00–0.88)	0.450
Rice	0.97 (0.37–2.06)	1.37 (0.86–2.20)	0.173	0.63 (0.20–1.16)	1.30 (0.60–2.18)	0.008
Noodles	1.30 (0.83–1.54)	1.25 (0.63–1.95)	0.735	0.63 (0.35–1.94)	1.23 (0.75–1.93)	0.025
Sweets	0.51 (0.24–0.78)	0.25 (0.07–0.42)	0.011	0.35 (0.19–0.91)	0.35 (0.09–0.73)	0.743
Potatoes	0.07 (0.00–0.12)	0.00 (0.00–0.08)	0.093	0.04 (0.00–0.14)	0.05 (0.00–0.11)	0.646
Fruits	1.64 (0.59–6.35)	1.30 (0.30–2.91)	0.159	1.44 (0.52–3.39)	1.25 (0.64–2.92)	0.882
Vegetables	1.85 (0.73–3.23)	1.32 (0.52–2.65)	0.333	1.51 (0.80–2.33)	1.14 (0.64–2.45)	0.935
Kimchies	0.84 (0.28–3.26)	1.43 (0.87–2.27)	0.940	0.85 (0.29–1.85)	0.98 (0.37–1.91)	0.795
Nuts	0.00 (0.00–0.07)	0.00 (0.00–0.00)	0.179	0.00 (0.00–0.10)	0.00 (0.00–0.01)	0.588
Legumes	0.41 (0.19–0.77)	0.36 (0.08–0.65)	0.288	0.40 (0.12–0.80)	0.32 (0.13–0.52)	0.721
Tea and coffee	0.46 (0.07–1.02)	0.39 (0.02–1.00)	0.699	0.21 (0.02–1.01)	0.43 (0.14–1.00)	0.383
Soda	0.43 (0.14–0.64)	0.43 (0.14–0.64)	0.939	0.04 (0.00–0.14)	0.21 (0.08–0.43)	0.008
Pastes	0.08 (0.03–0.14)	0.07 (0.03–0.14)	0.784	0.04 (0.02–0.14)	0.07 (0.02–0.14)	0.504
Animal fats	0.00 (0.00–0.05)	0.00 (0.00–0.03)	0.646	0.00 (0.00–0.02)	0.00 (0.00–0.02)	0.290
Dairy product	0.81 (0.31–1.47)	0.74 (0.27–1.31)	0.586	0.93 (0.29–1.73)	0.69 (0.33–1.64)	0.749
Eggs	0.40 (0.08–1.77)	0.29 (0.12–1.00)	0.771	0.43 (0.16–1.15)	0.57 (0.16–1.06)	0.671
Fishes and Shellfishes	0.28 (0.11–1.18)	0.12 (0.03–0.47)	0.071	0.16 (0.09–0.58)	0.30 (0.10–0.74)	0.217
Meats	1.04 (0.65–1.43)	0.86 (0.60–1.37)	0.511	0.53 (0.29–0.92)	0.50 (0.29–0.85)	0.929
Processed food	0.19 (0.07–0.25)	0.16 (0.12–0.29)	0.554	0.03 (0.00–0.18)	0.12 (0.03–0.23)	0.140
Fast food	0.47 (0.39–0.67)	0.42 (0.28–0.74)	0.487	0.30 (0.10–0.48)	0.28 (0.20–0.43)	0.353

^1^ Based on the classification of food categories presented by the Korean Rural Development Administration, foods were categorized into 20 food groups with some modifications. In consideration of the subject’s age, a fast food group containing hamburgers and pizza so on was added, and the grain group was subdivided into whole grains, rice, and noodles. ^2^ *p* values from Mann–Whitney U test. ^3^ Values are Median. The number in the bracket indicates first and third quartile. Median value of the serving size of the consumed food group has been adapted to the food intake recommendations and practices followed in KNHNES.

**Table 5:** Nutrient adequacy ratio (NAR) and mean adequacy ratio (MAR) of participants divided by gender.

		Men			Women	
	Depressive	Normal	*p*-Value ^1^	Depressive	Normal	*p*-Value ^1^
	(*n* = 18)	(*n* = 35)		(*n* = 21)	(*n* = 41)	
NAR ^2^						
Energy	0.69 (0.54–0.86) ^3^	0.90 (0.78–1.00)	0.003	0.79 (0.64–0.90)	0.92 (0.77–1.00)	0.017
Protein	1.00 (0.81–1.00)	1.00 (1.00–1.00)	0.003	1.00 (0.89–1.00)	1.00 (1.00–1.00)	0.038
Vitamin A	0.31 (0.19–0.51)	0.43 (0.31–0.61)	0.042	0.57 (0.37–0.77)	0.61 (0.51–0.85)	0.245
Thiamin	1.00 (0.95–1.00)	1.00 (1.00–1.00)	0.021	1.00 (0.87–1.00)	1.00 (0.98–1.00)	0.250
Riboflavin	0.81 (0.57–1.00)	0.96 (0.82–1.00)	0.183	1.00 (0.64–1.00)	1.00 (0.94–1.00)	0.207
Niacin	0.81 (0.58–1.00)	1.00 (0.82–1.00)	0.017	0.75 (0.59–0.95)	0.99 (0.81–1.00)	0.008
Vitamin B_6_	0.98 (0.77–1.00)	1.00 (0.95–1.00)	0.052	0.87 (0.65–1.00)	1.00 (0.82–1.00)	0.089
Folic acid	0.61 (0.41–0.92)	0.86 (0.63–1.00)	0.049	0.75 (0.49–1.00)	0.86 (0.67–1.00)	0.132
Vitamin B_12_	1.00 (1.00–1.00)	1.00 (1.00–1.00)	0.357	1.00 (0.84–1.00)	1.00 (1.00–1.00)	0.001
Vitamin C	0.34 (0.19–0.45)	0.37 (0.25–0.80)	0.128	0.46 (0.31–0.55)	0.61 (0.35–0.99)	0.119
Calcium	0.48 (0.31–0.65)	0.53 (0.36–0.68)	0.419	0.57 (0.38–0.71)	0.65 (0.49–0.83)	0.261
Phosphorus	1.00 (0.95–1.00)	1.00 (1.00–1.00)	0.019	1.00 (0.90–1.00)	1.00 (1.00–1.00)	0.102
Iron	1.00 (0.99–1.00)	1.00 (1.00–1.00)	0.475	0.97 (0.57–1.00)	1.00 (0.84–1.00)	0.320
Zinc	0.94 (0.70–1.00)	1.00 (0.93–1.00)	0.252	0.89 (0.72–1.00)	1.00 (0.83–1.00)	0.076
MAR ^4^	0.75 (0.65–0.88)	0.86 (0.78–0.93)	0.009	0.80 (0.64–0.92)	0.88 (0.80–0.94)	0.056

^1^ *p* values from Mann–Whitney U test. ^2^ NAR; nutrient adequacy ratio was calculated for a given nutrient is the ratio of subject’ intake to the current recommended nutrient intake for the subject’s sex and age category. ^3^ Median (first-third quartile). ^4^ MAR, mean of NAR was calculated by averaging NAR. NAR was truncated at 1 so that a nutrient with a high NAR could not compensate for a nutrient with a low NAR.

**Table 6:** Percentage of subjects below 75% of EAR ^1^ for each nutrient according to depression and gender.

		Men			Women	
	Depressive	Normal	*p*-Value ^2^	Depressive	Normal	*p*-Value ^2^
Nutrient	(*n* = 18)	(*n* = 35)		(*n* = 21)	(*n* = 41)	
Energy ^3^	10 (55.6) ^4^	8 (22.9)	0.017	9 (42.9)	7 (17.1)	0.028
Protein	2 (11.1)	0 (0.0)	0.044	2 (9.5)	2 (4.9)	0.481
Vitamin A	15 (83.3)	23 (65.7)	0.177	10 (47.6)	12 (29.3)	0.153
Thiamin	1 (5.6)	0 (0.0)	0.159	2 (9.5)	1 (2.4)	0.219
Riboflavin	5 (27.8)	3 (8.6)	0.064	5 (23.8)	1 (2.4)	0.007
Niacin	4 (22.2)	1 (2.9)	0.022	5 (23.8)	7 (17.1)	0.525
Vitamin B_6_	3 (16.7)	2 (5.7)	0.196	5 (23.8)	4 (9.8)	0.137
Folic acid	9 (50.0)	7 (20.0)	0.024	9 (42.9)	8 (19.5)	0.051
Vitamin B_12_	1 (5.6)	0 (0.0)	0.159	2 (9.5)	1 (2.4)	0.219
Vitamin C	15 (83.3)	23 (65.7)	0.177	18 (85.7)	20 (48.8)	0.005
Calcium	13 (72.2)	23 (65.7)	0.631	12 (57.1)	17 (41.5)	0.242
Phosphorus	1 (5.6)	1 (2.9)	0.625	3 (14.3)	1 (2.4)	0.072
Iron	1 (5.6)	1 (2.9)	0.625	5 (23.8)	5 (12.2)	0.239
Zinc	3 (16.7)	0 (0.0)	0.013	4 (19.0)	5 (12.2)	0.469

^1^ EAR: estimated average requirement. ^2^ *p* values from chi-square test. ^3^ Estimated energy requirements were used for calculation. ^4^ N (%).

## Data Availability

The data presented in this study are available on request from the corresponding author.
